# Breeding by intervening: Exploring the role of associations and deliberation in consumer acceptance of different breeding techniques

**DOI:** 10.1177/09636625231168087

**Published:** 2023-05-09

**Authors:** Paul Nales, Arnout R.H. Fischer

**Affiliations:** Wageningen University & Research, The Netherlands

**Keywords:** associations, consumer acceptance, deliberation, focus groups, gene-editing, genetic modification

## Abstract

New plant breeding techniques may play an important role in improving food quality, global food security and sustainability. Previous breeding techniques have, however, met with substantial resistance from society. This study examined the role of associations and deliberation in the evaluation of breeding techniques. Breeding techniques studied included conventional breeding, gene-editing, genetic modification (cisgenesis and transgenesis), marker-assisted breeding and synthetic biology. By using focus group discussions that included individual tasks, we found that when participants relied on their spontaneous associations, gene-editing was evaluated similarly as genetic modification. However, after information provision and group discussion, gene-editing was preferred over genetic modification. Perceived naturalness was found to be the main reason for obtaining different levels of acceptance, not only between gene-editing and genetic modification but across all breeding techniques examined. These findings highlight the importance of associations and show that beliefs about naturalness remain crucial in understanding how consumers evaluate breeding techniques.

## 1. Introduction

Global food security is under substantial pressure (Osendarp et al., 2022). It is estimated that in 2023, 8 to 13 million more people could become undernourished compared to 2022 (Food and Agriculture Organization (FAO), 2022). The fragility of the global food system has become more salient through the European droughts caused by climate change (Brás et al., 2021), the global COVID-19 pandemic and the 2022 war in Ukraine (Osendarp et al., 2022). Modern plant breeding, including gene-editing techniques, may play an important role in developing crops resilient to climate change, thereby improving food security when confronted with extreme weather conditions such as extreme drought (Shi et al., 2017). In addition, gene-editing techniques may also be of importance to increase food supply (Karavolias et al., 2021) and food quality (Kaur et al., 2020). The use of gene-editing techniques allows for precise modification, deletion or insertion of genes into an organism (Muringai et al., 2020). Previous genetic plant breeding techniques, most notably genetic modification, have however met with substantial resistance from consumers and society at large (Frewer et al., 2004). As a result, strict regulations were implemented by the European Union, as covered in ‘Regulation EC 1829/2003’ (The European Parliament and the Council of the European Union, 2003).

The distinction between genetic modification and gene-editing is particularly relevant since the European Court of Justice (2018) ruled in the *Confédération paysanne and Others v Premier ministre and Ministre de l’agriculture, de l’agroalimentaire et de la forêt* Case C528/16 that gene-editing is a form of genetic modification. This implies that the use of gene-editing for food production falls under the same strict regulations as genetically modified foods. Hence, according to the European Court of Justice, the European legislation does not differentiate between these different types of gene-based breeding, even though there are technical distinctions that separate gene-editing from earlier genetic modification techniques. It remains however unclear, to what extent the opinion of the European Court of Justice reflects the opinion of the public. That is, it remains unclear whether the public also perceives these techniques as similar or whether they perceive these techniques as distinctively different.

An important distinction between gene-editing and genetic modification is that certain subtypes of gene-editing can block specific genes without inserting exogenous DNA (Van de Wiel et al., 2017). The insertion of exogenous genes drives unnaturalness perception (Rozin, 2005), leading to a negative response (Debucquet et al., 2020). As a result, breeding techniques that insert exogenous genes are less accepted by consumers than conventional breeding techniques (Siegrist and Hartmann, 2020). The type of exogenous genes inserted also seems to be of importance. The insertion of genes from different species (transgenesis) is perceived more negatively compared to the insertion of genes from the same species, also referred to as cisgenesis (Delwaide et al., 2015). This suggests that the extent to which these techniques are accepted in part depends on the extent to which they intervene on genetic composition. Although there is some evidence that consumers perceive gene-editing and genetic modification to be similar (e.g. Shew et al., 2018), it also appears that consumers evaluate gene-editing techniques slightly more positively than genetic modification (Marette et al., 2021; Muringai et al., 2020; Ortega et al., 2022). These findings suggest that within society, there may be different levels of acceptance across genetic breeding techniques depending on the extent of intervention at the DNA level. It remains however unclear, to what extent levels of acceptance vary across different breeding techniques and whether genetic interventions without inserting exogenous DNA raise similar negative consumer perceptions as with genetic modification.

Prior research suggests that consumers have limited knowledge of breeding techniques and therefore often rely on quick responses that determine their attitude towards these techniques (Siegrist and Hartmann, 2020). Quick responses to a stimulus are often of an associative nature (Gawronski and Bodenhausen, 2006). In the context of this article, we define associations as the fast and automatic activation of interconnected concepts in the mind that are based on pre-existing linkages in memory (Gawronski and Bodenhausen, 2006). Associations contrast with deliberative, conscious responses which rely on active steering of thoughts (Evans, 2003). Research suggests that associations play an important role in consumer evaluations of genetically modified chocolate (Connor and Siegrist, 2011). Associations may thus also be important for evaluating foods created with new breeding techniques, especially for new techniques that appear similar to genetic modification, such as gene-editing. However, it remains unclear what associations consumers have with these new breeding techniques and to what extent these associations influence consumers’ acceptance of these techniques.

The aim of this study is to explore (1) whether new breeding techniques have different levels of acceptance compared to the current breeding techniques, (2) the role of associations in the evaluation of breeding techniques and (3) how these associative responses compare to deliberative responses. Studied techniques range from conventional breeding and gene-based selection techniques to genetic modification, gene-editing and synthetic biology. Specifically, we focus on how genetic modification compares to a (sub)type of gene-editing that solely blocks genes (site-directed nuclease 1 generations). To keep the distinction between gene-editing and genetic modification clear to participants, this study does not focus on other (sub)types of gene-editing that can insert exogenous genes such as site-directed nuclease 3 (Van de Wiel et al., 2017). Gaining insight into what associations consumers have and how these compare to deliberate perceptions may help us to better predict consumer responses and better understand the findings in the literature.

## 2. Theoretical framework

Consumers often rely on associative processes when evaluating a product, person or situation (Gawronski and Bodenhausen, 2006). Associations reflect the spontaneous activation of mental concepts that represent similarities between the perceived stimuli and the available representations in memory (Gawronski and Bodenhausen, 2011). We thus define associations as the spontaneous activation of concepts related to the perceived stimulus. Activated concepts may trigger feelings, which are then used to form an evaluation (Finucane et al., 2000). Activated concepts can be expressed in words to describe the mental representations that are associated with the perceived stimulus (Hoemann et al., 2019). Words and concepts associated with genetic modification often generate negative feelings, which in turn negatively influence evaluations (Connor and Siegrist, 2011). A similar effect has been found with synthetic biology, a new breeding technique based on creating genes from scratch (Steurer, 2016).

Associations can be semantically and conceptually related to the presented topic. Semantic associations are related to representation of the topic in words, signs and symbols (Minton et al., 2017). Conceptual associations are associations related to the meaning of the topic (Minton et al., 2017). The phrase *gene-editing* appears to be both conceptually and semantically related to genetic modification. Semantically, the similarity between ‘genetic’ from genetic modification and ‘gene’ from gene-editing will likely activate similar associations. The words ‘modification’ and ‘editing’ are not semantically similar; however, both words represent a concept of (human) intervention that may activate conceptually similar associations.

Associations are highly context-dependent (Gawronski and Bodenhausen, 2006). Different contexts may lead to the activation of different associations, which in turn could lead to a different evaluation. For example, applications of genetic modification in medicine are more accepted than for food purposes (Gaskell et al., 1999). Acceptance of genetic breeding techniques for food purposes is in part product-specific, where applying genetic modification to processed foods is more acceptable than to natural foods (Tenbült et al., 2005). Food products, serving as context, may thus play an important role in the associations retrieved, the feelings elicited and the resulting evaluation.

While initial associations may play an important role in shaping judgement, they are not necessarily decisive in the final evaluation. The extent to which associations are activated and influence the final judgement depends on the amount of deliberation people use when making a judgement (Gawronski and Bodenhausen, 2006). Deliberation enables people to re-assess the validity of retrieved associations through both recategorization and the development of inferences (Gawronski and Bodenhausen, 2006). Deliberation can be stimulated by information provision (Gawronski and Bodenhausen, 2011) and discussion between individuals (Gergen, 1985; Stoker et al., 2016). The new information, either provided or created through exchange of opinions and ideas, leads to additional associations and inferences which are then used to adjust evaluations. When few initial associations are available, additional associations and inferences may have substantial impact on evaluations. This explains why providing information about the benefits of genetic modification significantly affects willingness to pay for genetically modified foods (Lusk et al., 2004). Providing information about different genetic breeding techniques may thus stimulate deliberation, which enables consumers to retrieve new associations, develop new inferences and categorise gene techniques based on their distinctive properties. Hence, deliberative evaluations may deviate from associative evaluations, depending on the extent to which initial and later activated associations are validated.

In summary, associations are expected to be important in the evaluation of new breeding techniques due to semantic and conceptual similarity between associations retrieved from memory. Similar associations may lead to similar evaluations. Associations are context-dependent and therefore the food product type may influence acceptance. Finally, deliberative reasoning may lead to different evaluations compared to association-based evaluations.

## 3. Method

### Design

Six face-to-face focus groups (*n* = 6–8 each) were conducted, two each in three European countries (the Netherlands, Italy, and Czech Republic), to gain insights in the range of associations with and deliberations on breeding techniques. The countries represented the North-Western, Mediterranean and Central European regions. Each focus group was conducted in the local language and moderated by a native speaker. Focus groups were audio recorded after consent of all participants. The study received ethical approval from the Social Science Ethics Committee of Wageningen University & Research.

Focus groups were structured into three stages based on whether the tasks were individual or on a group level. The first stage was on an individual level and contained three tasks to collect individual data prior to any influence by group members in the discussion (a similar approach as used in Berezowska et al., 2014). The individual tasks were (1) an individual associative evaluation task, (2) an individual reading task and (3) an individual ranking task. The individual associative evaluation task was used to examine participants’ associations prior to any group discussion. The reading task was included to ensure that participants had sufficient knowledge for the focus group discussion in stage 2. The ranking task was used to gain insights in participants’ preferences that were discussed during the focus group discussion. The second stage was on a group level and consisted of the focus group discussion that followed a semi-structured focus group discussion guide/protocol. We chose to conduct focus groups as a group task as focus groups can stimulate deliberation through discussion between individuals in a natural way (Stoker et al., 2016). Finally, the third stage of the study was again on an individual level and consisted of another individual (final) evaluation task. Structuring the focus groups in this way allowed for collection of initial individual associations of participants (stage 1) while also triggering deliberation in the form of social discussion between members of the public (stage 2). In addition, insights in the stability of associative evaluations were obtained by having a final individual evaluation at the end of the focus group (stage 3). Each task is described in detail in the materials section.

### Participants

A total of 45 participants were recruited by marketing recruitment agencies across three European countries: the Netherlands (*n* = 16; 50% male, mean age = 42 (range = 18–71)), Italy (*n* = 16; 50% male, mean age = 42 (range = 20–62)), and Czech Republic (*n* = 13; 46% male, mean age = 38 (range = 19–63)). To prevent participants from searching for information before the start of the focus group, they were told that the topic would be food-related but were not informed that the discussion would be about breeding techniques used for food/crop production. For each focus group, quotas were set for gender, age and education to create a diverse group that represents a broad set of views. We limited the number of participants with food allergies and/or dietary preferences to a maximum of two participants per focus group session, to prevent personal concerns becoming a majority view in the discussions. Participants were rewarded according to the agreements made with the marketing recruitment agencies in the different countries.

### Materials and tasks

#### Stage 1: Associative evaluation task

The associative evaluation task was used to measure the range of participants’ individual spontaneous associations with, and evaluations of, four overarching breeding techniques: (1) genetic modification, (2) cross-pollination, (3) gene-editing and (4) synthetic biology. As we did not want to provide information at this stage, the distinction between cis and trans-genetic modification techniques was omitted. A booklet was prepared in which each individual participant wrote down the words that spontaneously came to mind when thinking of each breeding technique, with a maximum of 10 words per technique. Subsequently, participants selected one of the reported words which best reflected their concept of each breeding technique. Participants evaluated each word on how that word made them feel on a 5-point scale ranging from ‘very negative’ to ‘very positive’. Finally, participants provided an (associative) overall evaluation of the breeding technique on another 5-point scale. Together, two associative evaluation scores were obtained: (1) the evaluation of each (association-based) word mentioned and (2) the (associative) overall evaluation of each breeding technique. This process was repeated 4 times such that each breeding technique was evaluated. Because we were interested in spontaneous associations that would directly come to mind, participants were given a time limit of 15 minutes in total. Pre-test revealed that booklets were understandable but that many people had no associations with the term *conventional breeding*. As pre-test participants did had associations with cross-pollination, we used the wording cross-pollination instead of conventional breeding. The booklet can be found in the online Supplemental materials.

#### Stage 1: Information leaflets

After finishing the associative evaluation task, participants received information leaflets. The leaflets consisted of information about the breeding techniques that were discussed during the focus group discussion. Participants received information leaflets which started with general information about DNA and genes, followed by information of six breeding techniques (see Supplemental materials for all information provided). The final leaflet contained a ranking form which had to be used for the ranking task that followed the reading task. The following six breeding techniques were described on the leaflets: (1) cisgenesis (variant of genetic modification), (2) transgenesis (variant of genetic modification), (3) conventional breeding, (4) CRISPR-Cas9 (subtype of gene-editing), (5) synthetic biology (insertion of synthetically composed genes) and (6) marker-assisted breeding (non-invasive breeding tool). We specified cisgenesis (where genes from sexually compatible species are inserted) and transgenesis (where foreign genes from other species are used) as variants of genetic modification given these techniques substantially differ from each other and are regularly compared genetic modification techniques in social sciences (Beghin and Gustafson, 2021). Participants were instructed to carefully read the information and told the techniques would be used for the ranking task and focus group discussion that followed. The information was described as neutral as possible. Texts were structured and only differed where techniques were different from one another. Images explaining each breeding technique were added to support understanding of the techniques by participants. Participants were given up to 20 minutes to read the information but were allowed to start the ranking task once finished reading. The information leaflets remained available throughout the rest of the study.

#### Stage 1: Ranking task

Participants were asked to individually rank the six breeding techniques that were described on the information leaflets, based on their personal preferences. Specifically, participants had to assign each of the six breeding techniques to one of the following three groups: most preferred, moderately preferred or least preferred breeding technique. For each group, two techniques had to be assigned such that all techniques were assigned to only one group. This allowed for ties between techniques that might be perceived similar, and avoided the need to explain minor differences in perception within groups (which would be less manageable for a difference between all six options; see Berezowska et al., 2014). Participants were given 15 minutes for the ranking task.

#### Stage 2: Focus group discussion guide

The second stage of the study consisted of the focus group discussion. The focus group discussion followed a semi-structured protocol that was designed to (1) explore the range of perceptions, deliberations and preferences towards different breeding techniques through group discussion; (2) raise group discussion about the likes and dislikes of different breeding techniques and (3) have a group discussion on the implementation of different breeding techniques in food products to explore the practical relevance and, given the contextual dependency of associations, whether acceptance is in part product-specific.

#### Stage 3: Final evaluation task

The final evaluation task aimed to gain insights into the stability of the associative evaluation and to what extent evaluations changed due to the information provided and the discussion raised. The final evaluation task was an individual task that consisted of an evaluation form to evaluate the six breeding techniques discussed. On the evaluation form, participants had to evaluate each technique one final time on a 5-point scale ranging from ‘very negative’ to ‘very positive’.

All materials were prepared in English and translated into local languages by a native speaker. Pre-tests confirmed all materials (the booklet containing the associative evaluation task, the information leaflets, the ranking task and the final evaluation form) to be understandable. The English version of the booklet, the information leaflets, the ranking task form and the final evaluation form can be found in the online Supplemental materials.

### Procedure

Participants were welcomed to the focus group and offered refreshments. The moderator and participants introduced themselves by mentioning their name and, as icebreaker, their favourite dish. Thereafter, the moderator discussed the ground rules and provided participants an informed consent form. The informed consent form included consent for participation, recording and anonymised data collection and was to be signed if agreed. Participants were then handed out the booklet and asked to complete the associative evaluation task. This was followed by a short break after which participants were provided with the information leaflets about the six breeding techniques. When finished reading the information leaflets, participants did the ranking task. Thereafter, the focus group discussion started (stage 2), which lasted approximately 1 hour. After the discussion, participants were asked to make a final evaluation of the breeding techniques discussed (stage 3). Finally, participants were thanked for their participation, debriefed and booklets were collected. The entire procedure lasted approximately 1.5 hour.

### Data analysis

All associations mentioned in the individual booklets were translated into English and summarised in two ways. First, frequency tables were created for the words that were literally written down with each breeding technique. A cut off point of at least three words was used as indication of generally occurring associations, given fewer words would capitalise too much on individual opinions (cf. Fischer et al., 2007). Second, because words may vary in abstraction or have similar meaning, all words were coded and grouped into 13 overarching categories. Since all mentioned words fit into 1 of the 13 categories, no cut-off point was used for the second analysis. From the booklets, means were calculated of (1) the associative overall evaluation, and (2) the mean score of all words evaluated for each breeding technique.

For the rankings, cross tabulations were calculated with the breeding techniques as rows and the rankings as columns. Audio recordings of the group discussions were transcribed verbatim and translated into English. Transcripts were coded into emergent codes using Atlas.Ti 9 (version 9.1.6.0). Full (anonymised) data can be accessed at https://doi.org/10.17026/dans-xbg-kkbr. Finally, means were calculated and reported as illustration of the final evaluation. Given the small sample size, no formal statistical analyses were deemed relevant.

## 4. Results

### Stage 1: Individual associations prior to group discussion

#### Most mentioned associations

When comparing the most frequently written down words between the three genetic breeding techniques (genetic modification, gene-editing and synthetic biology), all genetic breeding techniques appear to be associated with similar concepts. Six out of eight most mentioned words with gene-editing were similar to the most mentioned words with genetic modification. The majority of (most) mentioned words with genetic breeding techniques were related to the concepts of genetics, artificial, modification or research. In contrast, cross-pollination was most frequently associated with concepts related to nature. None of the most mentioned words with cross-pollination were related to the top mentioned words of gene-editing and genetic modification, except for the word *artificial*, which might have been due to a carry-over effect. Synthetic biology was associated with both the concepts of artificiality (artificial and unnatural) and nature (nature and animals). Table 1 provides an overview of associations per technique that were mentioned at least 3 times.

**Table 1.** Most mentioned associations.

No.	Genetic modification	Cross-pollination	Gene-editing	Synthetic biology
	*n* = 231	*n* = 193	*n* = 168	*n* = 156
1	Laboratory (10x)	Bees (18x)	DNA (8x)	Artificial (18x)
2	Research (8x)	Flowers (11x)	Technology (5x)	Unnatural (7x)
3	Artificial (7x)	Plants (9x)	Adapting (4x)	Research (6x)
4	DNA (6x)	Nature (7x)	Future (4x)	Future (5x)
5	Future (6x)	Cross breeding (3x)	Research (4x)	Laboratory (4x)
6	Study (6x)	Insects (3x)	Change (4x)	Nature (4x)
7	Modification (6x)	Pollen (3x)	Artificial (3x)	Animals (3x)
8	Innovation (5x)	Artificial (3x)	Genes (3x)	Chemistry (3x)
9	Change (5x)			Synthesis (3x)
10	Chemistry (3x)			
11	Genes (3x)			
12	Unnatural (3x)			
13	Other (mentioned once or twice – 163x)	Other (mentioned once or twice – 136x)	Other (mentioned once or twice – 133x)	Other (mentioned once or twice – 103x)

#### Categorised associations

When categorising all words written down, it appears that from the genetic breeding techniques, genetic modification was associated more frequently with food/agriculture compared to gene-editing and synthetic biology. In contrast to genetic modification and gene-editing, synthetic biology was less associated with the concept of modification. Furthermore, gene-editing elicited substantially less associations in total compared to genetic modification and raised more associations with complexity and with DNA/genes. In contrast to the genetic breeding techniques, cross-pollination was highly associated with concepts related to nature. In addition, participants hardly associated cross-pollination with DNA/Genes, science, or with the concept of modification. Categorised associations with synthetic biology once more showed that synthetic biology was associated with both artificiality and nature. An overview of all categories and their frequency of being associated with each breeding technique is shown in Table 2.

**Table 2.** Thematically categorised associations (sorted by total number across all techniques).

No.	Code	GM (*n* = 231)	% of GM	CP (*n* = 193)	% of CP	GE (*n* = 168)	% of GE	SB (*n* = 156)	% of SB	Total (*N* = 748)	% of total
1	Nature	5x	2	82x	42	8x	5	21x	13	116	16
2	Modification	32x	14	7x	4	32x	19	10x	7	81	11
3	Science	38x	16	4x	2	16x	10	19x	12	77	10
4	Food/Agriculture	25x	11	28x	14	8x	5	7x	5	68	9
5	Artificial	16x	7	6x	3	5x	3	39x	25	66	9
6	Technology/Future	26x	11	8x	4	16x	10	16x	10	66	9
7	Improvement	15x	7	13x	7	14x	8	9x	6	51	7
8	Production	19x	8	11x	6	5x	3	11x	7	46	6
9	DNA/Genes	15x	7	1x	1	28x	17	1x	1	45	6
10	Risk/Health	10x	4	7x	4	11x	6	3x	2	31	4
11	Complexity	5x	2	5x	2	12x	7	2x	1	24	3
12	Laboratory	10x	4	2x	1	2x	1	5x	3	19	2
13	Other	15x	7	19x	10	11x	6	13x	8	58	8

GM: genetic modification; CP: cross-pollination; GE: gene-editing; SB: synthetic biology.

#### Associative evaluation

When comparing the means of the overall associative evaluations, cross-pollination was evaluated most positively. Furthermore, results show a negligible difference in evaluation between genetic modification and gene-editing (mean genetic modification of 3.24 vs mean gene-editing of 3.26). A similar pattern is shown when comparing the average evaluation of all the words written down per breeding technique (mean genetic modification of 3.48 vs mean gene-editing of 3.49). Synthetic biology received the lowest associative evaluation. An overview of the mean associative evaluation and the mean score of all words per breeding technique is shown in Table 3.

**Table 3.** Mean (standard deviation) associative evaluation and mean (standard deviation) evaluation of all words mentioned by participants.

	Genetic modification		Cross-pollination		Gene-editing		Synthetic biology	
	*M* (*SD*)	*n*	*M* (*SD*)	*n*	*M* (*SD*)	*n*	*M* (*SD*)	*n*
Mean overall associative evaluation	3.24 (1.026)	45	3.64 (0.609)	45	3.26 (0.828)	45	3.07 (0.846)	45
Mean score of all evaluated words per breeding technique	3.48 (1.156)	211	3.77 (0.869)	175	3.49 (0.865)	150	3.35 (1.115)	135

Because some words written down were not evaluated, the number of observations of mean scores of all evaluated words per breeding technique does not fully correspond with the number of mentioned associations in Tables 2 and 3.

#### Ranking task

**Figure 1.**
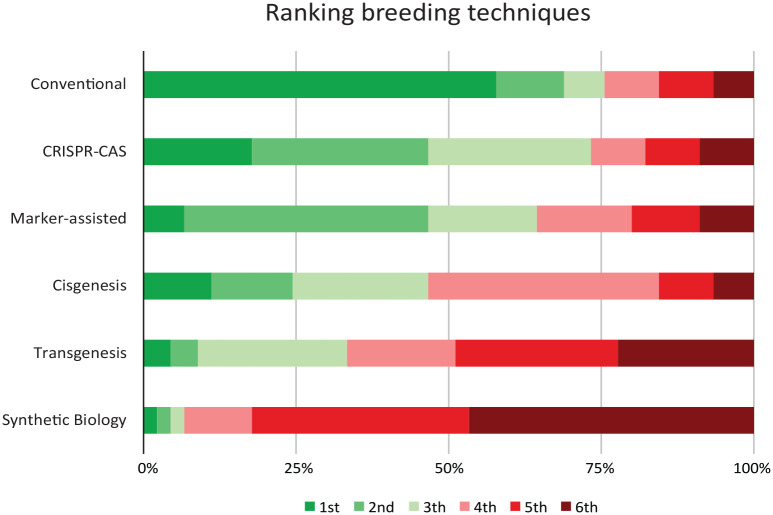
Overview: Ranking breeding techniques.

Results from the ranking task show patterns of different levels of acceptance across breeding techniques, with most participants preferring conventional breeding over the genetic breeding techniques (Figure 1).

### Stage 2: Focus group discussion

#### Focus group discussion: Reasoning behind ranking task

Across all participants, 58% ranked conventional breeding as most preferred breeding technique. The reasoning behind this was that in almost every case, conventional breeding was perceived as the most natural, or least invasive, method:

I had it [conventional breeding] on top because it is the most natural, without any manipulation. Actually, ah well, just as it always was. (Netherlands Focus Group 2, Participant 2)

Associative responses were also observed when participants explained their reasoning for preferring conventional breeding. Some participants explained how the words associated with conventional breeding influenced their perception and therefore their preference:

It [conventional breeding] is less invasive of nature also possibly . . . that is . . . the words [conventional breeding] are closer to us. The other words [genetic breeding techniques], as terms, are distant and even [conventional breeding] gives more the idea of pollination by bees, as to say like flowers in history. Additionally, to this we also use the word conventional [Translation note: in Italian “naturale” i.e., natural], it helps to assimilate the concept. (Italy Focus Group 2, Participant 6)

When participants deviated from the majority and preferred genetic breeding techniques over conventional breeding, their preferences were based on multiple reasons. Some participants perceived greater opportunities with genetic breeding techniques compared to conventional breeding, whereas others focused on perceived disadvantages of conventional breeding:

By exclusion, I would have put [conventional breeding] as an average, but the others inspired me more. It is nothing new, nothing advanced, all the others are new techniques. (Italy Focus Group 1, Participant 8)

But on the other side, from a breeder perspective, it is a problem that conventional breeding takes so much time. (Netherlands Focus Group 1, Participant 7)

A breeding technique that stood out in terms of ranking was marker-assisted breeding. In general, participants evaluated marker-assisted breeding positively; however, it was rarely selected as the most preferred breeding technique. Perceptions of marker-assisted breeding were related to research and controlling risks, which was perceived beneficial. Moreover, participants highly valued marker-assisted breeding being non-invasive. Yet, marker-assisted breeding was clearly perceived as an additional tool rather than a breeding technique, which was the primary reason for not ranking it as the most preferred technique:

In my opinion it [marker-assisted breeding] is a great technology, but it seemed to me to have little to do with the others where there is talk of an alteration of plant genes, while this is a technology that could be used together with the others, but which in itself does not change anything. (Italy Focus Group 2, Participant 6)

Regarding the least preferred breeding techniques, participants were clear. Approximately half the participants ranked synthetic biology as least preferred breeding technique and 83% of participants ranked synthetic biology in the lower bracket. The reasoning behind this was mostly similar to the reasoning for ranking conventional breeding as most preferred. That is, synthetic biology was perceived as the most invasive and least natural breeding technique:

It [synthetic biology] is too invasive for the plant and for us. (Italy Focus Group 1, Participant 3)

Participants’ perceptions were often strengthened by their conflicting associations with the semantics of synthetic biology. According to participants, the words *synthetic* and *biology* represent opposite concepts. Specifically, focussing on each word in isolation, the associations participants had with ‘biology’ were related to nature, whereas the associations with ‘synthetic’ were related to artificiality. Responses by participants indicated that combining these concepts creates tension, leading to a negative evaluation:

It [synthetic biology] seems like a contrast: biology is the study of life and synthetic is an opposite juxtaposition. (Italy Focus Group 1, Participant 8)

##### Difference between gene-editing and genetic modification

Most participants preferred gene-editing over genetic modification, whereas approximately one-third preferred genetic modification over gene-editing or had no clear preference. The reasoning for preferring gene-editing techniques was similar to the reasoning for preferring conventional breeding methods. That is, gene-editing was perceived less invasive, less artificial and it is not expected to lead to the development of new species as it was described in terms of gene blocking:

I think this [CRISPR-Cas9] is the least tampering, the easiest modification in the way that they are trying to erase the bad genes that can affect that the food will spoil easily, as my colleague here said and so on. (Czech Republic Focus Group 1, Participant 2)

Most of the times when genetic modification was preferred over gene-editing, it was not due to a heuristic decision rule such as naturalness, but to negative associations with gene-editing. For some participants, the associations they had with gene-editing were related to scariness. Other participants had negative associations with the word *blocking*:

And somehow, uhm, is CRISPR also inside my head applied to people. That they use it [CRISPR-Cas9] on babies to remove things. That I believe is also CRISPR-Cas I think, so I have a really negative association with this. (Netherlands Focus Group 2, Participant 3)

Additional reasons for preferring genetic modification over gene-editing appeared to be similar to preferring genetic breeding techniques over conventional breeding. That is, a minority of participants preferred genetic modification over gene-editing due to opportunities, effectiveness and/or efficiency benefits:

I feel like it [cisgenesis] is less difficult than CRISPR-Cas, where you have to basically eliminate all of the bad qualities that you do not want. Because, like, how many of them would you have to delete in order to produce a good result? (Czech Republic Focus Group 2, Participant 3)

#### Food products

When asking participants about the implementation of different breeding techniques in food products, the level of acceptance varied depending on the type of food product. The exception holds for synthetic biology, which was perceived unacceptable regardless of the type of food product. Food categories that were mentioned to affect participants’ level of acceptance were (un)natural/processed, (un)healthy and/or hedonic food products. The application of genetic breeding techniques on products that were perceived as natural was deemed less acceptable compared to processed products. Natural products that were mentioned were products such as vegetables, fruits and eggs. For processed hedonic products that were perceived unhealthy participants showed greater acceptance of genetic breeding techniques: Hedonic products that were mentioned were products such as chips, pizza and cola.

‘Are there any food products where it matters more to know that they were made like this [genetic breeding techniques]? . . .’ Everyone: ‘yes’ . . . Participant: ‘Fruit, vegetables and dairy products’. (Italy Focus Group 1, All participants)

#### Cross-country differences

Although there was general agreement on most issues, some differences between the countries emerged. In line with Italian food culture, Italians raised taste as an important aspect:

This thing [CRISPR-Cas9] of artificially correcting the defects that nature imposes, is a remarkable thing and will also bring a quality product that will have a balanced taste at the table. (Focus Group Italy 1, Participant 7)

Participants from the Czech Republic attributed great value to the country of origin of the products they buy:

I always try to look where the product comes from. If there’s a possibility to buy a product from the Czech Republic, I will do that to support local products. (Czech Republic Focus Group 2, Participant 7)

Finally, the Dutch showed associations with the COVID-19 vaccine in both focus groups, whereas the Italians and Czechs did not mention anything related to COVID. This could have been influenced by the timing of the focus group (the Dutch group ran in parallel to the first mass vaccination in the Netherlands), or due to the associations that Dutch participants have with genetic modification and the COVID-19 vaccine, such as long-term risk effects:

A lot of testing etc. . . And it probably can go wrong on the long term, you do not know that either. Just like with, yes with the Corona vaccine, for example. You do not know that either. So that will take time. . . I guess? (Netherlands Focus Group 1, Participant 3)

### Stage 3: Individual final evaluation

**Figure 2.**
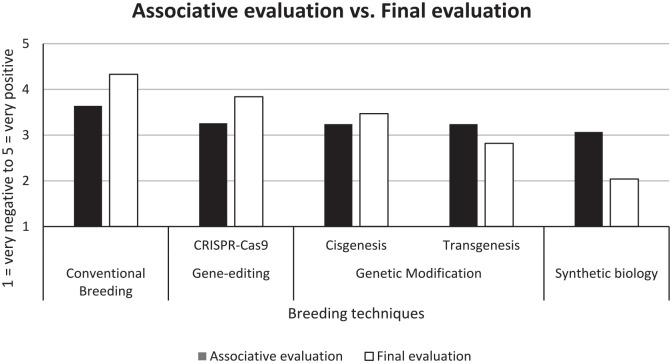
Mean associative evaluation versus mean final evaluation. Because we used genetic modification in the associative task, cisgenesis and transgenesis are rated as identical in associative evaluation. Marker-assisted breeding was not included in the associative evaluation and therefore the final evaluation of marker-assisted breeding is not presented in this figure.

When comparing the final evaluation with the associative evaluation, we observe different evaluations. Both conventional breeding and gene-editing were evaluated more positively. Cisgenesis was evaluated slightly more positively compared to the primary associative evaluation of genetic modification, whereas transgenesis and synthetic biology were evaluated less positively than their associative response. Figure 2 provides an overview of the differences between the associative and final (deliberative) evaluation.

## 5. Discussion

This study shows different levels of acceptance across a spectrum of breeding techniques. Perceived naturalness appears to be the main differentiation when evaluating different breeding techniques. This is in line with previous research that suggests naturalness to be an important heuristic when evaluating breeding techniques (Siegrist and Hartmann, 2020). In this study, participants specifically ranked the different breeding techniques against the single dimension of perceived naturalness. That is, the level of acceptance corresponded to the level of perceived naturalness for each breeding technique. This aligns with and contextualises findings of Schenk et al. (2011) who demonstrated that conventional breeding methods are preferred over genetic modification, with Delwaide et al. (2015) who showed a higher degree of consumer acceptance of cisgenesis over transgenesis, with Ortega et al. (2022) who showed a greater degree of acceptance for gene-editing compared to transgenesis, and with Muringai et al. (2020) who demonstrated a slightly lower willingness to pay for genetic modification compared to gene-editing.

A technique that stood out was marker-assisted breeding. Responses to marker-assisted breeding show that using DNA technology to select breeds based on genetic composition is not perceived negatively, even if the technology to do so is distinctly unnatural. We argue that the distinction lies in the fact that marker-assisted breeding is not invasive in the natural process of breeding itself. Hence, we argue that naturalness as a heuristic (Siegrist and Hartmann, 2020) is used by consumers as a relevant rule of thumb but only to consider the extent to which the intervention in the crop itself is perceived natural.

Prior research has shown that associations are of great importance in the evaluation of genetic modification (Connor and Siegrist, 2011). This study contributes by expanding the range of breeding techniques, thereby enabling comparison between associations with different breeding techniques and gaining insight into the role of associations in the evaluation of different breeding techniques. More specifically, our focus on associations provides insight on why naturalness is such a strong heuristic: Naturalness is already strongly present during the initial retrieval of associations. This implies that the first concepts that come to mind when thinking about breeding techniques are related to naturalness (or its opposite: artificiality). It therefore seems obvious to rely on naturalness as a heuristic when evaluating breeding techniques, even though the influence of naturalness may be inflated by availability bias (Folkes, 1988).

Activation and evaluation of conflicting associations influence opinions on breeding techniques. Breeding is related to crops, which in turn are associated with nature (Rozin et al., 2012). While crops are associated with nature, the genetic breeding techniques (genetic modification, gene-editing and synthetic biology) are associated with artificiality. Simultaneous activation of conflicting associations creates tension in the mind of participants and can lead to the unpleasant experience of ambivalence (Van Harreveld et al., 2015). In contrast, conventional breeding is associated with nature and therefore congruent with the associations with crops, leading to a preference for conventional breeding. Conflicting associations are most evident with synthetic biology. We argue that this is due to the importance of conceptual and semantic-based associations in consumer evaluations. Synthetic biology consists of two semantic elements: synthetic, which is conceptually associated with artificial, and biology, which is conceptually associated with nature. The importance of semantics can also explain why terms with a similar ‘gene’ word element as gene-editing and genetic modification generated similar associations and subsequently similar evaluations. In addition, conceptual associations with ‘modification’ and ‘editing’ may have led to the activation of similar concepts, thereby also increasing perceived similarity.

The perception of naturalness is more than a shallow association. Naturalness is a deeply rooted belief (Rozin et al., 2004), that is culturally bounded (Rozin et al., 2012). Beliefs about naturalness are particularly strong in the food domain (Rozin et al., 2004), especially regarding plants (Rozin et al., 2012). Based on our focus group discussions, we argue that naturalness as a belief is used as a decision rule because it provides meaning to the evaluation, where it serves as a reason for disliking invasive techniques. In addition, application of genetic breeding techniques on unprocessed food products was deemed more negative compared to the application of genetic breeding techniques on processed food products. This is in line with prior research showing different acceptance levels of genetic modification across a range of food products (Tenbült et al., 2005), where acceptance for more highly processed products was higher. We argue that this may be caused, at least in part, by the relationship between naturalness and healthiness. Natural foods are considered healthier than processed foods (Rozin et al., 2004). Breeding natural foods with genetic breeding techniques decreases the perceived naturalness of these natural foods and may thus also decrease healthiness perception. For food products that are ultra-processed and already perceived as unhealthy and unnatural, it appears to be less important which breeding technique is used.

The difference between associative evaluations and final evaluations shows the instability of initial judgements or attitudes when expressed without a substantial amount of knowledge (Armitage and Conner, 2000). Following information provision and focus group discussion, knowledge increased, and deliberation was stimulated, which mobilised greater use of cognitive resources to arrive at a stronger attitude. Except for gene-editing, we observed that cognitive resources were used to justify and thus reinforce the initial associative response. We argue that the initial associations with naturalness and artificiality determined the direction of reasoning. By deliberating on this initial response, participants enforced original associations. This is in line with prior research which suggests that when additional cognitive resources confirm the associative response, it strengthens the relationship between the associative and deliberative response (Gawronski and Bodenhausen, 2006). As a result, the initial evaluation of conventional breeding became more positive, whereas the initial evaluation of genetic modification and synthetic biology became more negative.

Opinions on gene-editing were different. They started with the same negative associations with artificiality as genetic modification, yet gene-editing was ultimately perceived as more positive. The deliberative line of reasoning that gene-editing is less invasive compared to genetic modification could be an explanation for this change from the initial attitude. Due to the information provided and the discussion raised, participants perceived gene-editing as less invasive compared to genetic modification. Given their initial limited knowledge of gene-editing, the new knowledge may have changed their relatively unstable concept of gene-editing, which appeared to be quite similar to genetic modification beforehand. Hence, deliberating on information and discussion caused participants to differentiate between techniques, which may have led to the accommodation of knowledge (Piaget, 2000). In contrast, the new knowledge obtained from the other breeding techniques may have fit within their existing initial associations, leading to the assimilation of knowledge, which in turn reinforced their existing views (Piaget, 2000). An alternative explanation could be a comparison effect. The deliberative line of reasoning that gene-editing is less invasive may have led participants to better differentiate between techniques. As a result, participants could differentiate between techniques based on invasiveness which may have facilitated contrast effects (e.g. Davidenko et al., 2015), leading to a more positive evaluation of gene-editing compared to genetic modification.

Our study also sheds some light on how consumers in general deliberate on topics of which they have limited knowledge. During deliberation, we observed that additional associations became active, which were then used to justify initial associative responses. These additional associations were only indirectly related to the information provided. Deliberation appeared to trigger a deeper exploration of the associative network until participants were sufficiently confident about their opinion based on these associations. That is, associations activated additional associations, which in turn triggered larger networks that include heuristics and beliefs learned from prior experience. As a result, more information was retrieved from memory, leading to greater attitudinal certainty. Hence, we argue that the deliberation process operated more as a meta-cognitive process steering towards certainty in associative response, rather than a separate, logic-based reasoning process on the topic in hand.

On a practical level, our findings show differences in acceptance across a spectrum of breeding techniques ordered on perceived naturalness and invasiveness. When using these techniques for societally relevant purposes such as climate change and global food security, it may be useful to consider using a less invasive technique to gain greater acceptance and less resistance. Furthermore, especially when advocating gene-editing, it is important to provide consumers with information on the technique. Due to the retrieval of semantically similar associations derived from the ‘gene’ word element and conceptually similar associations from the ‘editing’ word element, gene-editing and genetic modification are likely to be perceived similar when no information about these techniques is provided. This also stresses the importance of the naming of breeding techniques, as ‘unlucky’ name choice may set the public against these techniques (Boersma et al., 2019). Our findings contribute to the societal debate by showing that not all techniques may elicit equal resistance from the silent majority. As less invasive techniques are perceived to be more acceptable, it may be beneficial to make this explicit in the name such that different associations are retrieved.

For this study, we conducted six focus groups across three European countries. Although the sample size was limited, the reduced additional insights with each consecutive group suggest theoretical saturation regarding the range of perceptions. In all countries, we observed the same pattern in which a majority of participants mentioned naturalness as the most important driver for preferring conventional breeding over genetic breeding techniques. In addition, supporters of genetic breeding techniques also showed similar patterns in reasoning across countries. That is, supporters of genetic breeding techniques focused on either abstract benefits described as ‘opportunities’ or disadvantages of conventional breeding such as ‘time-consuming’. We found only minor differences in reasoning between countries such as the importance of taste (Italy), the country of origin (Czech Republic) and COVID associations (the Netherlands).

Providing information about multiple techniques and asking participants to rank these techniques may have influenced opinions and is thus a limitation of this study. In this study, such comparison was crucial to foster an appropriate discussion about the breeding techniques. In reality, consumers often do not have the information at hand and will often only consider one technique at a time. In addition, the exact contents of the information may have influenced the evaluation, although in this study the extent and direction in which the evaluation changed did not necessarily follow the information provided. To disentangle these explanations, in future research the effects of deliberation and information should be studied in isolation. Another limitation of the information provided is the number of variants used. We limited the discussion to six techniques, and for gene-editing we only included CRISPR-Cas9 with a focus on blocking genes. There are, however, many more techniques particularly in the emerging field of gene-editing, several of which can also insert exogenous DNA (e.g. site-directed nuclease 3 generations). We decided to exclude these options as we wanted to present the differences between a limited set of breeding techniques in such way that it would be understandable and manageable in the context of a focus group discussion without causing confusion between techniques. It is conceivable that including the insertion of genes as possibility for gene-editing would lead to different comparison with, in particular, genetic modification. Further study is needed to establish to what extent our findings reflect opinions on gene-editing in general, or that the difference between blocking and insertion within gene-editing options matters.

### Conclusion

Consumers show different levels of acceptance across a spectrum of breeding techniques. Associations and deliberation on information play an important role in both the evaluation of breeding techniques and in the extent to which consumers develop different levels of acceptance. When relying on associations, gene-editing and genetic modification are at first similar, while only with information provision and group discussion, consumers differ in acceptance of these techniques. After information and discussion, conventional breeding remains the most preferred option, followed by gene-editing, genetic modification and, finally, synthetic biology. These preferences rely on the perception that invasive techniques are unnatural and therefore disliked.

## Supplemental Material

sj-pdf-1-pus-10.1177_09636625231168087 – Supplemental material for Breeding by intervening: Exploring the role of associations and deliberation in consumer acceptance of different breeding techniques Click here for additional data file. Supplemental material, sj-pdf-1-pus-10.1177_09636625231168087 for Breeding by intervening: Exploring the role of associations and deliberation in consumer acceptance of different breeding techniques by Paul Nales and Arnout R.H. Fischer in Public Understanding of Science
