# Supplementary material for: Breeding by intervening: Exploring the role of associations and deliberation in consumer acceptance of different breeding techniques
Source: Public Underst Sci. 2023 May 9;32(7):889–906. doi: 10.1177/09636625231168087 (PMC10552337; doi:10.1177/09636625231168087)
Supplement: sj-pdf-1-pus-10.1177_09636625231168087 – Supplemental material for Breeding by intervening: Exploring the role of associations and deliberation in consumer acceptance of different breeding techniques [file sj-pdf-1-pus-10.1177_09636625231168087.pdf]

Supplemental Materials

Breeding by intervening:

Exploring the role of associations and deliberation in consumer acceptance of different breeding techniques

Paul Nales, Arnout R. H. Fischer

Marketing and Consumer Behaviour Group, Social Sciences, Wageningen University & Research

Table of contents:

1. **Booklet associative evaluation task**
2. **Information leaflets**
3. **Ranking task form**
4. **Final evaluation form**

1. Booklet Associative evaluation task

Technology: Genetic Modification

	Step 1	Step 2	Step 3				
#	Please write down the words that first come to mind when you think of Genetic Modification	Which word best describes Genetic Modification?	How do you feel about each word that came to mind?				
1			O Very negative	O Negative	O Neutral	O Positive	O Very positive
2			O Very negative	O Negative	O Neutral	O Positive	O Very positive
3			O Very negative	O Negative	O Neutral	O Positive	O Very positive
4			O Very negative	O Negative	O Neutral	O Positive	O Very positive
5			O Very negative	O Negative	O Neutral	O Positive	O Very positive
6			O Very negative	O Negative	O Neutral	O Positive	O Very positive
7			O Very negative	O Negative	O Neutral	O Positive	O Very positive
8			O Very negative	O Negative	O Neutral	O Positive	O Very positive
9			O Very negative	O Negative	O Neutral	O Positive	O Very positive
#			O Very negative	O Negative	O Neutral	O Positive	O Very positive

Step 4				
How do you feel about Genetic Modification?				
O Very negative	O Negative	O Neutral	O Positive	O Very positive

Technique: Cross pollination

	Step 1	Step 2	Step 3				
#	Please write down the words that first come to mind when you think of Cross pollination	Which word best describes Cross pollination?	How do you feel about each word that came to mind?				
1			O Very negative	O Negative	O Neutral	O Positive	O Very positive
2			O Very negative	O Negative	O Neutral	O Positive	O Very positive
3			O Very negative	O Negative	O Neutral	O Positive	O Very positive
4			O Very negative	O Negative	O Neutral	O Positive	O Very positive
5			O Very negative	O Negative	O Neutral	O Positive	O Very positive
6			O Very negative	O Negative	O Neutral	O Positive	O Very positive
7			O Very negative	O Negative	O Neutral	O Positive	O Very positive
8			O Very negative	O Negative	O Neutral	O Positive	O Very positive
9			O Very negative	O Negative	O Neutral	O Positive	O Very positive
#			O Very negative	O Negative	O Neutral	O Positive	O Very positive

Step 4				
How do you feel about Cross pollination?				
O Very negative	O Negative	O Neutral	O Positive	O Very positive

Technology: Gene-editing

#	Step 1<br>Please write down the words that first come to mind when you think of Gene-editing	Step 2<br>Which word best describes Gene-editing?
1		
2		
3		
4		
5		
6		
7		
8		
9		
#		

Step 3				
How do you feel about each word that came to mind?				
O Very negative	O Negative	O Neutral	O Positive	O Very positive
O Very negative	O Negative	O Neutral	O Positive	O Very positive
O Very negative	O Negative	O Neutral	O Positive	O Very positive
O Very negative	O Negative	O Neutral	O Positive	O Very positive
O Very negative	O Negative	O Neutral	O Positive	O Very positive
O Very negative	O Negative	O Neutral	O Positive	O Very positive
O Very negative	O Negative	O Neutral	O Positive	O Very positive
O Very negative	O Negative	O Neutral	O Positive	O Very positive
O Very negative	O Negative	O Neutral	O Positive	O Very positive
O Very negative	O Negative	O Neutral	O Positive	O Very positive

Step 4				
How do you feel about Gene-editing?				
O Very negative	O Negative	O Neutral	O Positive	O Very positive

Technology: Synthetic Biology

#	Step 1<br>Please write down the words that first come to mind when you think of Synthetic Biology	Step 2<br>Which word best describes Synthetic Biology?
1		
2		
3		
4		
5		
6		
7		
8		
9		
#		

Step 3				
How do you feel about each word that came to mind?				
O Very negative	O Negative	O Neutral	O Positive	O Very positive
O Very negative	O Negative	O Neutral	O Positive	O Very positive
O Very negative	O Negative	O Neutral	O Positive	O Very positive
O Very negative	O Negative	O Neutral	O Positive	O Very positive
O Very negative	O Negative	O Neutral	O Positive	O Very positive
O Very negative	O Negative	O Neutral	O Positive	O Very positive
O Very negative	O Negative	O Neutral	O Positive	O Very positive
O Very negative	O Negative	O Neutral	O Positive	O Very positive
O Very negative	O Negative	O Neutral	O Positive	O Very positive
O Very negative	O Negative	O Neutral	O Positive	O Very positive

Step 4				
How do you feel about Synthetic Biology?				
O Very negative	O Negative	O Neutral	O Positive	O Very positive

2. **Information leaflets**

General information

Plants and other living beings are made up of cells.

Cells can divide themselves; a cell can split in two. This is how plants grow, how they make leaves, flowers and fruits.

At the core of each cell are chromosomes. Chromosomes are made up of DNA and genes. The DNA contains the hereditary properties and characteristics of the plant.

Within the DNA are smaller pieces of information. These smaller pieces are called "genes". Each gene affects a particular trait.

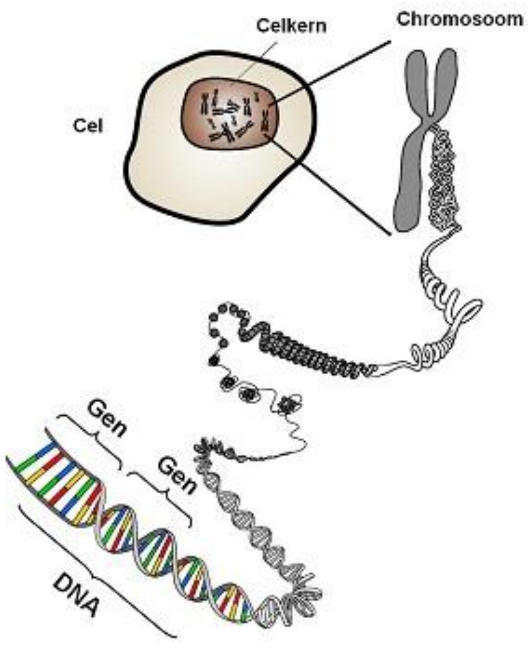

If we look through a strong microscope, DNA looks like a long strand of double helices. A piece of DNA is called a gene.

Conventional Breeding

Conventional breeding is a breeding technique that develops new plants by using cross pollination.

With cross pollination, a plant is fertilized by the pollen from the **same species of plant**.

This leads to the development of seeds. The seeds contain **genes from both plants.**

As a result, new generations of plants grown from the seeds will show characteristics of both plants.

Which characteristics are inherited depends on which genes are exchanged during fertilization.

With conventional breeding, we can bring **random properties** into a plant that come from **plants of the same species.**

Globally:

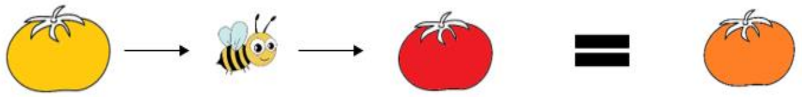

On DNA level:

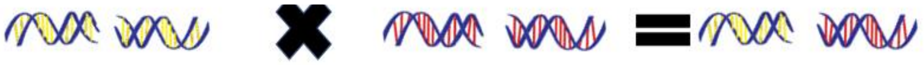

Genes:
Genes are small pieces of DNA that contain certain hereditary characteristics.

DNA:
DNA is located at the core of each cell and contains all hereditary characteristics and properties of a plant.

Cell:
Plants and all other living beings are made up of cells. A cell is the smallest part of a plant.

CRISPR-CAS

CRISPR-CAS is a breeding technique that develops new plants.

With CRISPR-CAS, unwanted **genes from the plant** are **blocked**.

As a result, the plant is no longer able to show the characteristics caused by the blocked genes.

With CRISPR-CAS we can **remove specific properties** from a plant.

Globally:

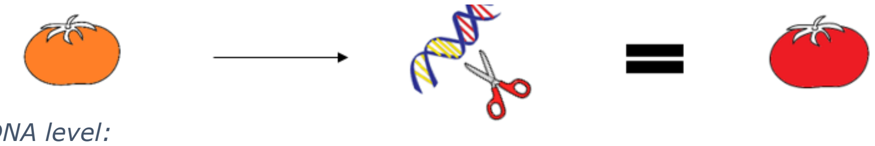

On DNA level:

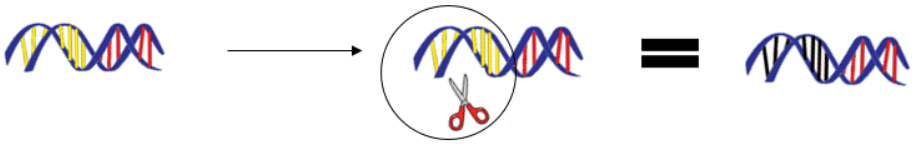

Genes:
Genes are small pieces of DNA that contain certain hereditary characteristics.

DNA:
DNA is located at the core of each cell and contains all hereditary characteristics and properties of a plant.

Cell:
Plants and all other living beings are made up of cells. A cell is the smallest part of a plant.

Transgenesis

Transgenesis is a breeding technique that develops new plants.

With transgenesis a gene of the plant is swapped for a **gene from an unrelated organism**.

As a result, the plant loses one of its own genes and gets a gene from an unrelated organism instead.

The added gene causes the plant to show a new characteristic.

With transgenesis we can bring **specific properties** into a plant that come from **unrelated organisms**.

Globally:

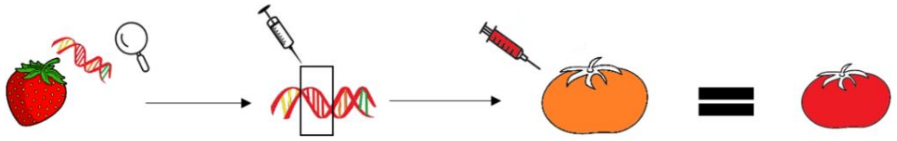

On DNA level:

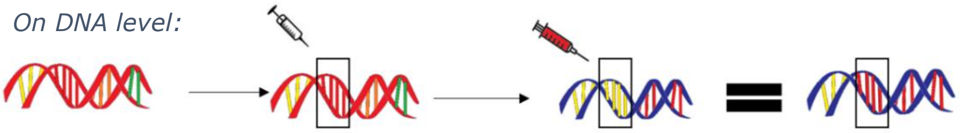

Genes:
Genes are small pieces of DNA that contain certain hereditary characteristics.

DNA:
DNA is located at the core of each cell and contains all hereditary characteristics and properties of a plant.

Cell:
Plants and all other living beings are made up of cells. A cell is the smallest part of a plant.

Cisgenesis

Cisgenesis is a breeding technique that develops new plants.

With cisgenesis a gene of the plant is swapped for a **gene from a related plant species**.

As a result, the plant loses one of its own genes and gets a gene from a related plant species instead.

The added gene causes the plant to show a new characteristic.

With cisgenesis we can bring **specific properties** into a plant that come from **related plant species**.

Globally:

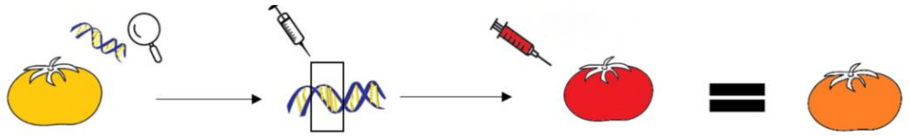

On DNA level:

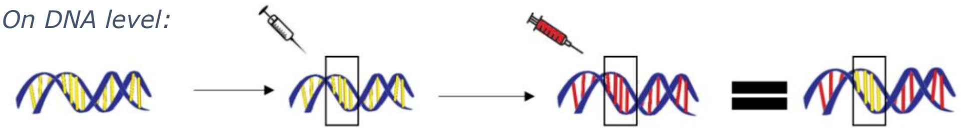

Genes:
Genes are small pieces of DNA that contain certain hereditary characteristics.

DNA:
DNA is located at the core of each cell and contains all hereditary characteristics and properties of a plant.

Cell:
Plants and all other living beings are made up of cells. A cell is the smallest part of a plant.

Marker assisted breeding

Marker assisted breeding is a breeding technique used for developing new plants.

Marker assisted breeding **analyses the DNA of the plant**.

This makes it possible to see which genes cause certain properties of the plant.

New generations of plants can then be analysed to see if they also contain the genes of interest.

If the new generations contain those genes, then they also contain the desired properties.

With marker assisted breeding, we can select **plants with the properties of interest**.

Globally :

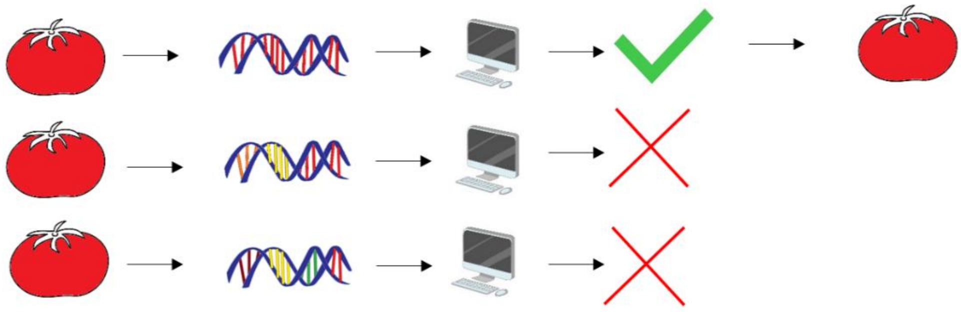

Genes:
Genes are small pieces of DNA that contain certain hereditary characteristics.

DNA:
DNA is located at the core of each cell and contains all hereditary characteristics and properties of a plant.

Cell:
Plants and all other living beings are made up of cells. A cell is the smallest part of a plant.

Synthetic Biology

Synthetic Biology is a breeding technique that develops new species of plants.

With synthetic biology a gene of the plant is swapped for an **artificially composed gene**.

As a result, the plant loses one of its own genes and gets an artificially composed gene instead.

The added gene causes the plant to show a new characteristic.

With synthetic biology we can bring **specific properties** into a plant that come from **artificially composed genes**.

Globally:

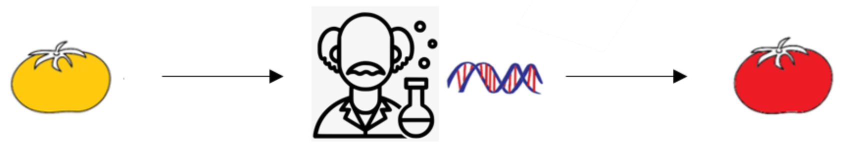

On DNA level:

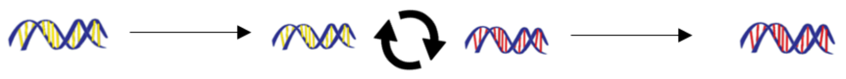

Genes:
Genes are small pieces of DNA that contain certain hereditary characteristics.

DNA:
DNA is located at the core of each cell and contains all hereditary characteristics and properties of a plant.

Cell:
Plants and all other living beings are made up of cells. A cell is the smallest part of a plant.

3. Ranking task form

Technologies:
Synthetic Biology<br>Cisgenesis<br>Marker-assisted Breeding<br>Conventional Breeding<br>CRISPR-CAS<br>Transgenesis

Most preferred technologies	1	
	2	

Middle	1	
	2	

Least preferred technologies	1	
	2	

4. Final evaluation form

#	Technology	Wat is your opinion about each technology?				
1	Conventional Breeding	O Very negative	O Negative	O Neutral	O Positive	O Very positive
2	CRISPR-CAS	O Very negative	O Negative	O Neutral	O Positive	O Very positive
3	Transgenesis	O Very negative	O Negative	O Neutral	O Positive	O Very positive
4	Cisgenesis	O Very negative	O Negative	O Neutral	O Positive	O Very positive
5	Marker-assisted Breeding	O Very negative	O Negative	O Neutral	O Positive	O Very positive
6	Synthetic Biology	O Very negative	O Negative	O Neutral	O Positive	O Very positive
